# First *LDLRAP1* and Recurrent *LDLR* Mutations in Tunisian Families With Familial Hypercholesterolemia

**DOI:** 10.1111/jcmm.70997

**Published:** 2026-01-05

**Authors:** Wirath Ben Ncir, Afif Ben‐Mahmoud, Hamdi Frikha, Fatma Abdelhedi, Faten Hadj Kacem, Nabila Majdoub, Mouna Mnif, Hyung‐Goo Kim, Leila Ammar Keskes, Jouke‐Jan Hottenga

**Affiliations:** ^1^ Laboratory of Human Molecular Genetics Faculty of Medicine of Sfax Sfax Tunisia; ^2^ Neurological Disorder Research Center, Qatar Biomedical Research Institute (QBRI) Hamad Bin Khalifa University (HBKU), Qatar Foundation Doha Qatar; ^3^ Department of Endocrinology Hedi Chaker Hospital of Sfax Sfax Tunisia; ^4^ Department of Neurosurgery, Robert Wood Johnson Medical School, Rutgers The State University of New Jersey Piscataway New Jersey USA

**Keywords:** ADH, ARH, autosomal dominant hypercholesterolemia, autosomal recessive hypercholesterolemia, familial hypercholesterolemia, *LDLR*, *LDLRAP1*

## Abstract

Familial hypercholesterolemia (FH) is a genetic disorder characterised by elevated plasma LDL‐cholesterol, predisposing to premature atherosclerotic cardiovascular disease. Most cases follow an autosomal dominant pattern (ADH) caused by pathogenic variants in *LDLR, APOB or PCSK9*. In contrast, the rare autosomal recessive form (ARH) results from biallelic mutations in *LDLRAP1*, leading to defective LDL receptor‐mediated endocytosis. Despite the high rate of consanguinity in Tunisia, *LDLRAP1* variants have not yet been reported in this population. In this study, Whole Exome Sequencing of two consanguineous Tunisian families, identified distinct pathogenic variants. In the first family (FH‐A), a recurrent *LDLR* splice‐site variant (c.1845+1G>A) was detected in both heterozygous and homozygous states, consistent with an autosomal dominant inheritance pattern. In the second family (FH‐B), a novel homozygous *LDLRAP1* missense variant (c.161G>A; p.Gly54Asp) was identified, confirming autosomal recessive inheritance. *In silico* analyses using MutationTaster, DynaMut2, MUpro, DDGun, NetSurfP‐2.0, ConSurf and PyMOL predicted that the p.Gly54Asp substitution destabilises the PTB domain of LDLRAP1 by disrupting key hydrogen bonds and hydrophobic interactions, thereby likely impairing LDLR internalisation. According to ACMG guidelines, this variant is classified as likely pathogenic. Clinically, ARH patients exhibited early‐onset xanthomas and an unusual quadricuspid aortic valve (QAV). Targeted analysis of valvulogenesis genes (*NOTCH1, GATA4, NKX2‐5, TBX5, AGTR1, BMP2*) revealed no co‐segregating pathogenic variants, suggesting that QAV may result from embryonic LDL accumulation disrupting *Notch1* signalling rather than a monogenic defect. Comparison with other ADH Tunisian families carrying the same *LDLR* mutation showed phenotypic variability, likely influenced by genetic modifiers, treatment response and environmental factors. These findings provide the first evidence of *LDLRAP1*‐associated ARH in Tunisia and highlight the genetic heterogeneity of FH, emphasising the importance of integrating molecular, structural and functional analyses for accurate diagnosis, personalised management and early prevention.

## 1 Introduction

Familial hypercholesterolemia (FH) is an inherited disorder characterised by elevated serum low‐density lipoprotein cholesterol (LDL‐C), leading to premature atherosclerosis and cardiovascular disease. Excess LDL‐C leads to cholesterol deposition in peripheral tissues (xanthomas) and accelerates atherosclerosis, promoting early‐onset coronary heart disease [1]. FH is primarily caused by pathogenic variants in genes regulating LDL metabolism—*LDLR, APOB, PCSK9*, and, more rarely, *LDLRAP1* [2]. Among these, *LDLR* mutations account for nearly 90% of cases, typically inherited in an autosomal dominant manner [3].

At the molecular level, the *LDLR* gene encodes the low‐density lipoprotein receptor, a transmembrane glycoprotein responsible for binding and internalising circulating LDL particles via apolipoprotein B. Once internalised through clathrin‐coated pits, the receptor‐ligand complex undergoes endocytosis, followed by dissociation in the endosome and subsequent recycling of the receptor to the cell surface. *LDLRAP1* encodes a cytosolic adaptor protein essential for linking LDLR to the clathrin and AP‐2 complex, ensuring efficient LDL uptake [4, 5].

Autosomal recessive hypercholesterolemia (ARH) is caused by pathogenic variants in *LDLRAP1*, which disrupt LDL internalisation without altering the receptor structure, despite normal LDLR expression [6, 7]. In contrast, autosomal dominant hypercholesterolemia (ADH) results from mutations in *LDLR*, *APOB* or *PCSK9*, which directly impair LDL binding, ligand integrity or receptor availability at the cell surface. This defect typically results in plasma LDL‐C levels exceeding 500 mg/dL and confers a high risk of early‐onset complications, including atherosclerotic cardiovascular disease and aortic valve stenosis [4, 8]. Studies using patient‐derived induced pluripotent stem cell (iPSC)‐derived hepatocyte‐like cells have shown that LDLRAP1 deficiency only partially reduces LDL uptake, suggesting the existence of alternative internalisation pathways [9]. In addition to the classical FH‐associated genes, *ApoE* variants have also been implicated. *ApoE*, a key component of VLDL and chylomicrons, mediates hepatic lipoprotein uptake via receptors including *LDLR*. Pathogenic *ApoE* variants can disrupt this process, leading to a phenotype similar to autosomal dominant FH, overall contribution is relatively minor compared with the canonical FH genes [10, 11, 12].

Heterozygous familial hypercholesterolemia (HeFH) affects about 1 in 311 individuals, whereas the homozygous form (HoFH), caused by biallelic pathogenic variants in *LDLR, APOB*, *PCSK9 or LDLRAP1*, is far rarer, with an estimated prevalence of 1 in 250,000–360,000 [13]. The frequency varies by ethnicity, being highest in Black populations and lowest in Asian populations [3, 10, 14, 15, 16, 17]. Early diagnosis is crucial, as up to 90% of affected individuals remain undiagnosed and fewer than 1% receive adequate treatment [18]. Genetic testing allows accurate distinction between HeFH and HoFH, enabling timely initiation of intensive lipid‐lowering therapy, particularly in children at risk of premature cardiovascular disease [19].

**FIGURE 1:**
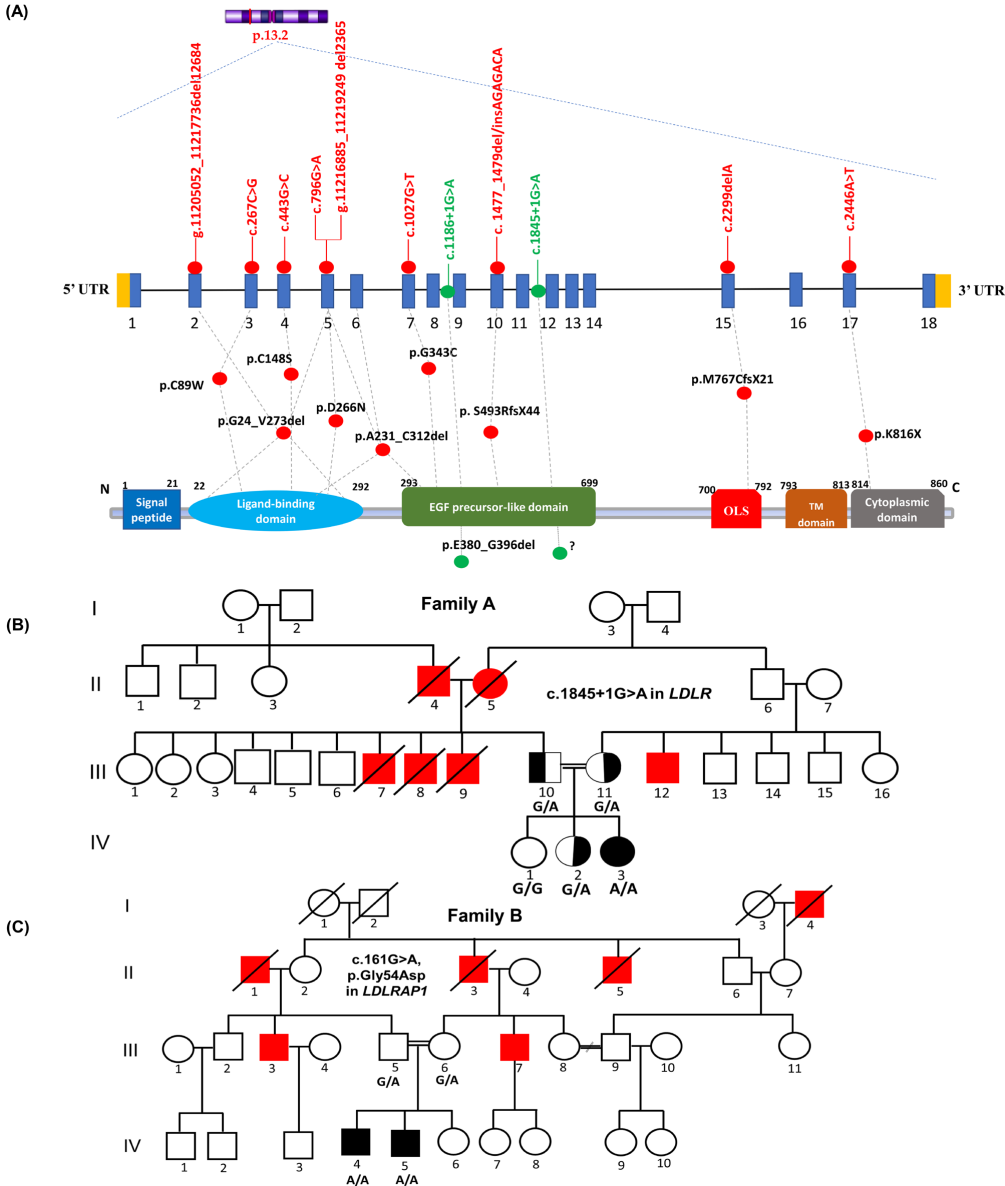
Genetic and clinical characterisation of Tunisian families with familial hypercholesterolemia. (A) Schematic representation of Reported *LDLR* Mutations in Tunisian Patients at both the cDNA and Protein Levels. The upper panel shows *LDLR* gene located at 19p13.2, illustrating the 11 intragenic mutations (red circles) and two splice‐site variants (green circles) mapped to their corresponding exons and introns (*LDLR*, NM_000527.5). Exons are depicted in blue boxes, introns as connecting black lines, and untranslated regions (UTRs) as yellow boxes. The lower panel illustrates the LDLR protein domain structure (NP_000518.1), including the signal peptide (exon 1), ligand‐binding domain (exons 2–6), EGF precursor‐like domain (exons 7–14), O‐linked sugar (OLS) domain (exon 15), transmembrane (TM) domain (exons 16 and 5′ part of exon 17), and the cytoplasmic domain (3′ region of exon 17 and 5′ region of exon 18). Red and green dots beneath the protein structure indicate the precise locations of identified variants. (B, C) Pedigrees of consanguineous Tunisian families A and B carrying *LDLR* and *LDLRAP1* variants, respectively. Red symbols indicate individuals diagnosed with cardiovascular disease (CVD) before the age of 50. A diagonal line denotes deceased individuals, and shaded symbols represent affected genotyped members.

In Tunisia, the prevalence of HeFH is estimated at approximately 1 in 165, significantly higher than the global average [20]. Nevertheless, systematic screening and molecular characterisation remain limited. To date, eleven *LDLR* and one *PCSK9* pathogenic variants have been reported, while no *LDLRAP1* variants have been identified (Figure 1A and Table 1) [21, 22, 23, 24, 25, 26, 27, 28]. This highlights a major gap in the understanding of genetic spectrum of FH within the Tunisian population.

**TABLE 1:** Reported molecular variants in *LDLR* and *PCSK9* among hypercholesterolemic individuals from Tunisia.

Exon/intron	cDNA variant	Protein variant	Variant type	References
*LDLR* gene (NM_000527.5 / NP_000518.1)				
2_5^a^	g.11205052_11217736del12685	p.Gly24_Val 273del	Major rearrangement	[21, 22]
3	c.267C>G	p.Cys89Trp	Missense	[21, 23]
4	c.443G>C	p.Cys148Ser	Missense	[21, 24]
5	c.796G>A	p.Asp266Asn	Missense	[24]
5_6^a^	g.11216885_11219249del2365	p.Ala231_Cys312del	Major rearrangement	[22]
7	c.1027G>T	p.Gly343Cys	Missense	[21, 23]
8–9	c.1186+1G>A	p.Glu380_Gly396del	Splice site	[21, 25]
10^a^	c.1477_1479del/insAGAGACA	p.Ser493ArgfsX44	Frame shift	[21, 23, 26]
11/12	c.1845+1G>A	?	Splice site	[21, 27]
15^a^	c.2299delA	p.Met767CysfsX21	Frameshift	[28]
17	c.2446A>T	p.Lys816X	Nonsense	[21, 23]
*PCSK9* gene (NM_174936.4; NP_777596.2)				
3^a^	c.520C>T	p.Pro174Ser	Missense	[28]

*Note:* ?″ reflects the absence of a simple protein consequence, although the variant is expected to disrupt LDLR protein production.

^a^ Mutations reported solely in the Tunisian population.

The present study aims to investigate the molecular basis of FH in two consanguineous Tunisian families, identify causative variants and inheritance patterns, and expand knowledge of FH genetics in North Africa. Tunisia's unique genetic diversity—shaped by centuries of admixture among Arab, Berber, European and Sub‐Saharan ancestries—may contribute to a distinctive distribution of FH‐associated variants, underscoring the need for population‐specific studies.

## 2 Material and Methods

### 2.1 Patients

Nine individuals from two unrelated Tunisian families (A and B) were analysed, including three probands and six first‐degree relatives. The pedigrees of families A and B are shown in Figure 1B,C, respectively.

**FIGURE 2:**
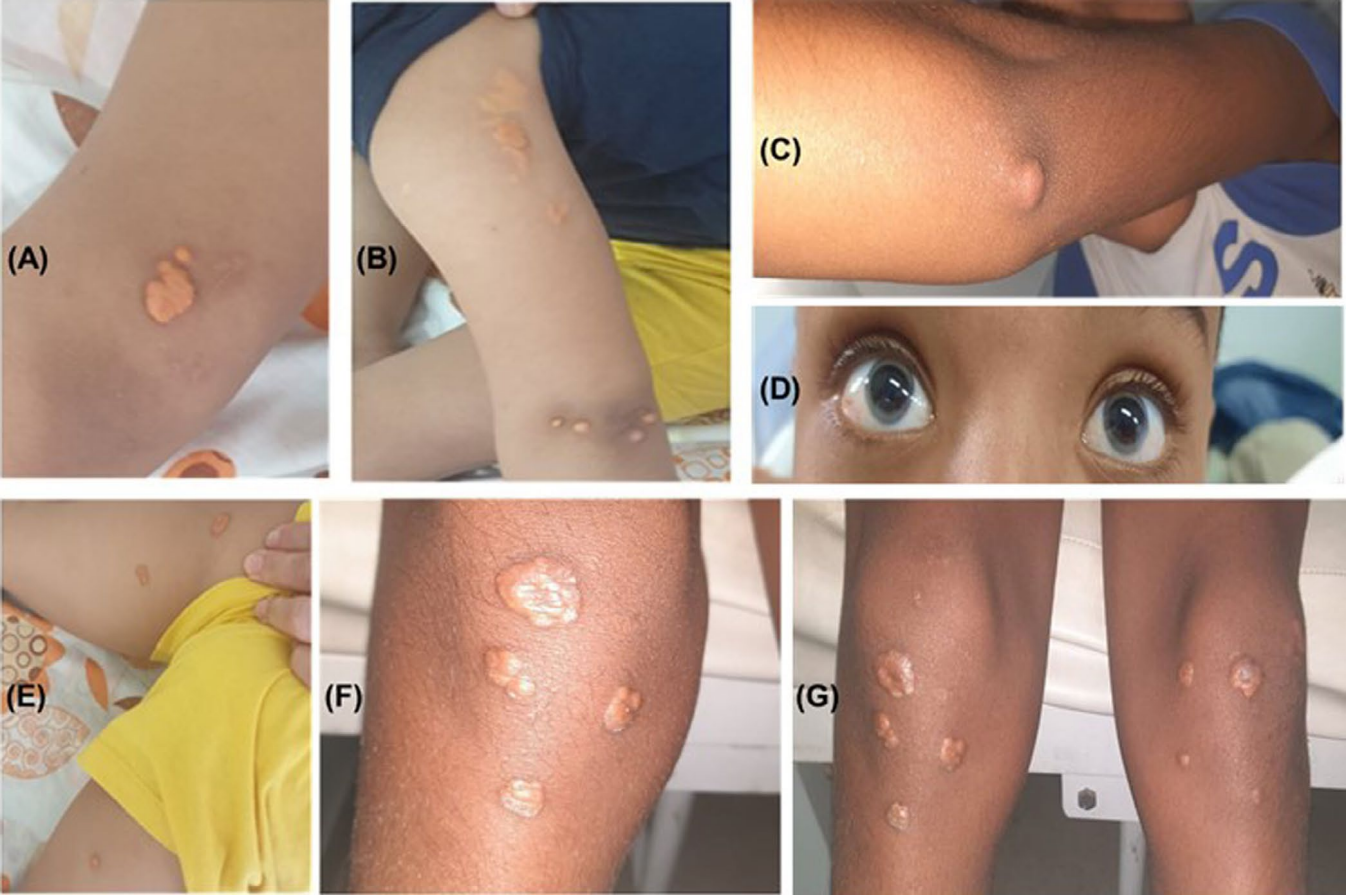
Clinical presentation of cutaneous and ocular manifestations in patients from Families A and B. Representative clinical features of affected individuals IV‐3 (Family A; panels A, B and E) and IV‐4 (Family B; panels C, F and G). Multiple well‐demarcated yellowish plaques and nodules consistent with xanthomas are visible on the elbows (A–C), axilla (B), groin (E) and knees (F, G). Subcutaneous nodules are present on the extensor surface of the arm (B). An ocular abnormality, corneal arcus, is evident in both eyes of patient IV‐4 (D).

The probands were diagnosed with FH based on established clinical criteria, including markedly elevated plasma levels of total cholesterol and low‐density lipoprotein cholesterol (LDL‐C > 8 mmol/L), a family history of hypercholesterolemia, and the presence of characteristic dermatological signs such as tuberous xanthomas (particularly on the elbows and knees), tendon xanthomas and corneal arcus (gerontoxon). These cutaneous signs were first noted during dermatological examinations (Figure 2).

Further evaluation revealed diffuse carotid artery infiltration with atheromatous plaques in the right common carotid artery. Personal and/or familial histories of premature cardiovascular disease were also documented. Secondary causes of hypercholesterolemia, including diabetes mellitus, hypothyroidism and nephrotic syndrome, were systematically excluded.

Detailed family and medical histories were obtained for each subject following informed consent. The families were selected based on the Dutch Lipid Clinic Network (DLCN) criteria, a widely used tool for the clinical diagnosis of FH. This method integrates clinical and family histories, physical examination and LDL‐C levels. A DLCN score of > 6 was considered indicative of probable or definite FH, in accordance with current international guidelines [3, 13]. The lipid profile, additional clinical diagnoses and treatment regimens of the patients are summarised in Table 2.

**TABLE 2:** Lipid profile, additional clinical diagnosis, and treatment regimen of the patients in this study.

Family ID	Code	Sex	Age	TC mmol/L	TG mmol/L	LDL‐C mmol/L	HDL‐C mmol/L	Tuberous xanthomas	Tendinous xanthomas	Corneal acrus	CHD	Treatment
A	III‐10	M	−	5.9	1.7	4.3	0.8				+	Atorvastatin 80 mg/day+ ezetemibe 10 mg/day
	III‐11	F	−	9.3	1.43	7.3	1.33				−	Atorvastatin 20 mg/day
	IV‐1	F									−	Rosuvastatin 5 mg/day
	IV‐2	F	8 years	8.7	0.67	7.08	1.31				−	−
	IV‐3	F	6 years	28.4	1.6	26.37	1.3	+	+	+	−	Atorvastatin 10 mg/day
B	III‐5	M	—	Normolipidemic								
	III‐6	F	—	Normolipidemic								
	IV‐4	M	12 years	7.4	0.78	5.75	1.3	+	+	+	−	Rosuvastatin 10 mg/day
	IV‐5	M	9 years	8.47	0.8	6.90	1.21		+	+	−	Rosuvastatin 10 mg/day

Abbreviations: CHD, Coronary Heart Disease; TC, Total Cholesterol; TG, Triglycerides.

### 2.2 Genetic Analysis and Predicting of Variant Pathogenicity

Genomic DNA was extracted from frozen whole‐blood samples collected in EDTA tubes using the standard phenol–chloroform method. DNA quantification was then performed using a Qubit fluorometer, and its purity was assessed using a NanoDrop spectrophotometer. Following quality validation, Whole Exome Sequencing (WES) was performed on DNA samples from all individuals included in this study, as previously described [29].

The bioinformatics pipeline for WES data analysis involved sequential steps of variant filtration and prioritisation. Analysis was restricted to exonic regions and canonical splice sites regions. Variants with a minor allele frequency (MAF) < 1% in population databases (gnomAD) were retained. Variants were then prioritised according to the known involvement of the corresponding gene in familial hypercholesterolemia and the observed co‐segregation of the variant with the disease within affected families. All candidate variants were cross‐referenced with dbSNP and ClinVar to identify previously reported variants and to determine any known clinical significance.

Variants interpretation followed ACMG guidelines and classified as pathogenic, likely pathogenic, variant of uncertain significance (VUS), likely benign, or benign [30]. Candidate variants implicated in FH causation were subsequently confirmed by Sanger sequencing. Specific primers were designed to amplify exon 2 of *LDLRAP1* and exon 12 of *LDLR* via polymerase chain reaction (PCR) [30]. Primer sequences and annealing temperatures are provided in Table S1. PCR products were sequenced using an Applied Biosystems Genetic Analyser, and forward DNA strands of the targeted exons were analysed.

To evaluate the functional consequences of candidate variants, several *in silico* prediction tools were used, including MutationTaster, Meta‐SNP, CADD and MutPred2. MutationTaster assesses both coding and non‐coding variants by integrating evolutionary conservation, splice‐site alterations, protein features and known disease mutations. Meta‐SNP is a meta‐predictor that combines outputs from PANTHER, PhD‐SNP, SNAP and SIFT to generate a consensus pathogenicity score. MutPred2 predicts the structural and functional effects of non‐synonymous substitutions using sequence‐based, structural and evolutionary parameters. CADD (Combined Annotation Dependent Depletion) integrates multiple genomic annotations to provide a Phred‐like score ranking variants by their likelihood of pathogenicity.

### 2.3 Comparative Analysis of Amino Acid Conservation

The reference protein sequence of LDLRAP1 was retrieved from UniProt (accession number Q5SW96). For structural reference, the Protein Data Bank (PDB) entry corresponding to the LDLR cytoplasmic tail bound to the phosphotyrosine‐binding (PTB) domain of LDLRAP1 was obtained from the RCSB PDB database (accession number 3SO6).

Comparative sequence analysis of LDLRAP1 orthologs across multiple species was performed to evaluate amino acid conservation. Reference sequences were retrieved from Ensembl (https://www.ensembl.org) for *Danio rerio* (zebrafish), *Takifugu rubripes* (fugu), *Xenopus tropicalis* (frog), *Mus musculus* (mouse), *Gallus gallus* (chicken), *Felis catus* (cat), *Macaca mulatta* (monkey), *Pan troglodytes* (chimpanzee) and *Homo sapiens* (human) (Figure 4A).

### 2.4 Evaluation of Protein Stability and Dynamics

To assess the effects of amino acid substitutions on protein stability and dynamics, three computational tools were employed: *DynaMut2*, *MUpro* and *DDGun* [31, 32]. *DynaMut2* applies normal mode analysis (NMA) to estimate variant‐induced changes in protein flexibility and stability, providing graphical visualisation of conformational alterations [33]. *MUpro* integrates both sequence‐based and structure‐based predictive models to evaluate the impact of variants on protein folding, stability and function. *DDGun* combines sequence‐derived features and evolutionary conservation data to predict the effects of single amino acid substitutions on protein stability. Collectively, these tools enhance the interpretation of structural consequences of variants and support the identification of potentially pathogenic variants.

### 2.5 Structural Analysis and Functional Prediction

To further evaluate the structural impact of the identified pathogenic variant, several computational tools were employed. *NetSurfP‐2.0* was used to predict secondary structure, residue disorder and solvent accessibility at the mutation site. This tool employs an advanced architecture combining convolutional neural networks (CNNs) and long short‐term memory (LSTM) networks for highly accurate structural predictions [34]. *AlphaFold2*, was applied to predict the three‐dimensional (3D) conformation of proteins with high accuracy. The *AlphaMissense* extension of *AlphaFold2* was used to predict the potential pathogenicity of missense variants [35]. Additionally, *ConSurf* was used to assess evolutionary conservation of residues at and around the mutation site [36].

### 2.6 Protein–Protein Interactions and Structural Properties Analysis

To investigate the interactions between LDLRAP1 and LDLR, the protein complex structure (PDB ID: 3SO6) was retrieved from the Protein Data Bank. Using the *PyMOL* mutagenesis wizard, the specific variant was introduced at the relevant residue within the phosphotyrosine‐binding (PTB) domain of LDLRAP1. *PyMOL* tools were then employed to compare the binding interfaces and interactions between LDLRAP1 and LDLR in both the wild‐type and mutant structures. Structural alterations, including changes in β‐sheet and α‐helix elements, were analysed and visualised using *PyMOL* graphics software to generate detailed figures illustrating these differences [37].

## 3 Results

### 3.1 Identification of the c.1845+1G>A Mutation in *LDLR* in Tunisian Family A

**FIGURE 3:**
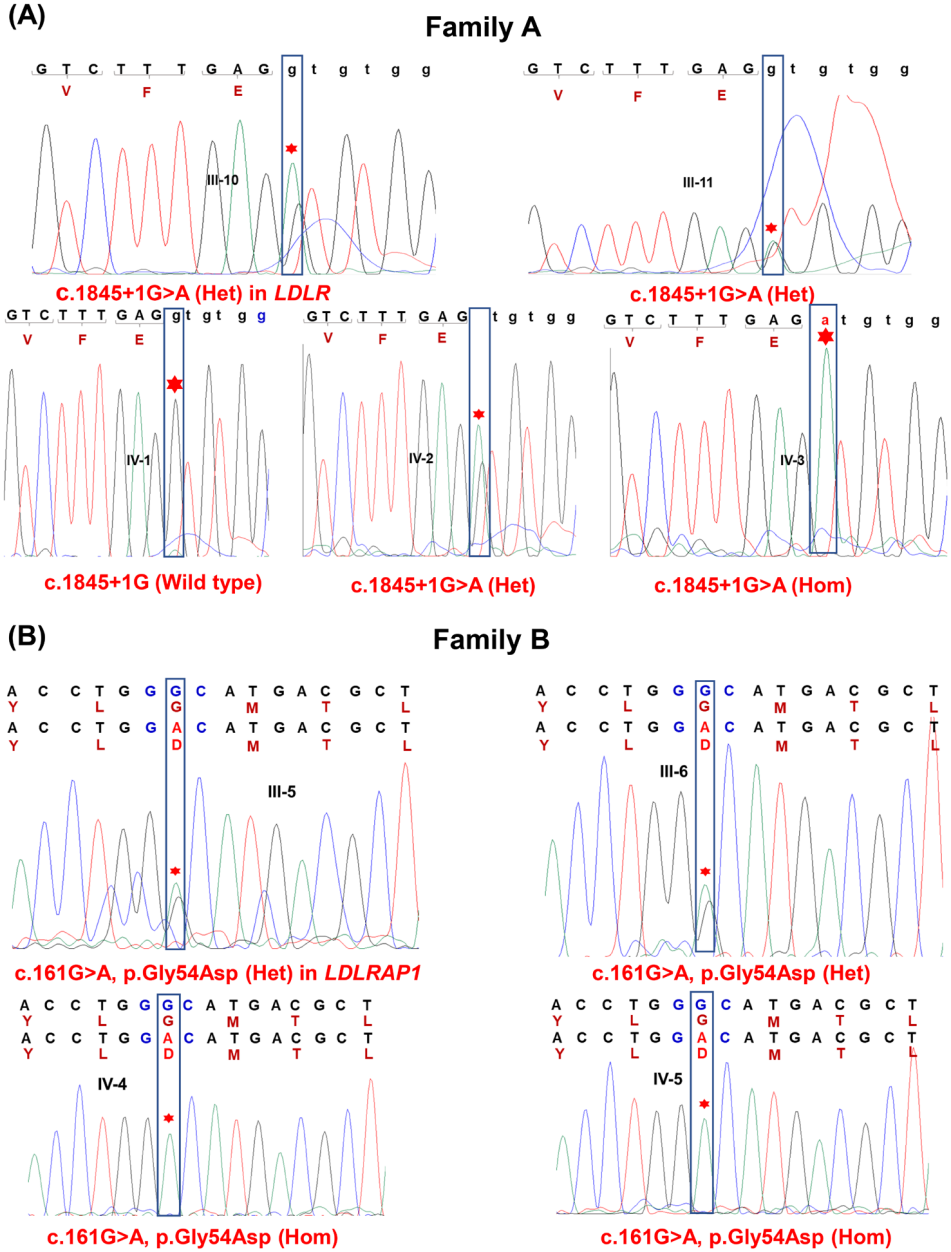
Sanger sequencing chromatograms showing wild‐type and mutant alleles of *LDLR* and *LDLRAP1* in Tunisian families with familial hypercholesterolemia. Red asterisks indicate the positions of the nucleotide substitutions. (A) Splice‐site mutation c.1845+1G>A in *LDLR* identified in Family A. Individuals III‐10 and III‐11 (parents) are heterozygous carriers; IV‐1 (unaffected daughter) is homozygous wild‐type; IV‐2 is heterozygous; and IV‐3 (affected proband) is homozygous for the variant. Lowercase letters represent intronic sequences, uppercase letters denote exonic sequences, and the corresponding amino (in brown) are shown below the exonic codons. (B) Missense variant c.161G>A (p.Gly54Asp) in *LDLRAP1* identified in Family B. Above each chromatogram, the wild‐type (top) and mutant (bottom) nucleotide and amino acid sequences are displayed. Individuals III‐5 and III‐6 (unaffected parents) are heterozygous carriers, while IV‐4 and IV‐5 (affected brothers) are homozygous for the variant. Variant nomenclature follows HGVS recommendations.

WES identified a rare co‐segregating variant in the *LDLR* gene in the first Tunisian family with FH (Family A): c.1845+1G>A, corresponding to a guanine‐to‐adenine substitution at the +1 position of intron 12. This splice‐site variant alters the highly conserved GT dinucleotides at the 5′ donor site to AT, a change predicted to disrupt normal mRNA splicing. Sanger sequencing revealed that the affected proband (IV‐3) from family A is homozygous for the *LDLR* splice‐site variant c.1845+1G>A mutation in intron 12 (NM_000527.5; Figures 1B and 3A). The mother (III‐11), father (III‐10) and sister (IV‐2), all affected, are heterozygous carriers, whereas the unaffected sister (IV‐1) carried no variant allele (Figures 1B and 3A). Segregation analysis is consistent with an autosomal dominant inheritance in this family.

### 3.2 Identification of the c.161G>A Variant in *LDLRAP1* in Tunisian Family B

WES performed on four members from the second Tunisian family with (family B) identified a novel co‐segregating missense variant, c.161G>A, in exon 2 of the *LDLRAP1* gene (NM_015627.3) (Figures 1C and 3B). This variant was present in the homozygous state in the two affected brothers (IV‐4 and IV‐5) and in the heterozygous state in their unaffected consanguineous parents (III‐5 and III‐6) (Figures 1C and 3B).

The *LDLRAP1* c.161G>A variant results in Gly54Asp amino acid substitution (NP_056442.2). This residue is located within the N‐terminal PTB domain, which mediates the interactions of LDLRAP1 with the FDNPxY motif in the cytoplasmic tail of the LDL receptor and with phosphoinositides at the cell membrane. Functionally, LDLRAP1 also associates with clathrin and its adapter AP‐2 via its C‐terminus sequences, thereby facilitating LDLR internalisation through clathrin‐mediated endocytosis.

Based on the ACMG criteria, this variant was classified as likely pathogenic. According to multiple *in silico* prediction tools (see Methods), the p.Gly54Asp substitution is predicted to impair protein stability and function. Sanger sequencing confirmed the segregation pattern observed in the WES analysis (Figures 1C and 3B).

### 3.3 Evolutionary Conservation of the Gly54 Residue in *LDLRAP1* Across Vertebrate Orthologs

**FIGURE 4:**
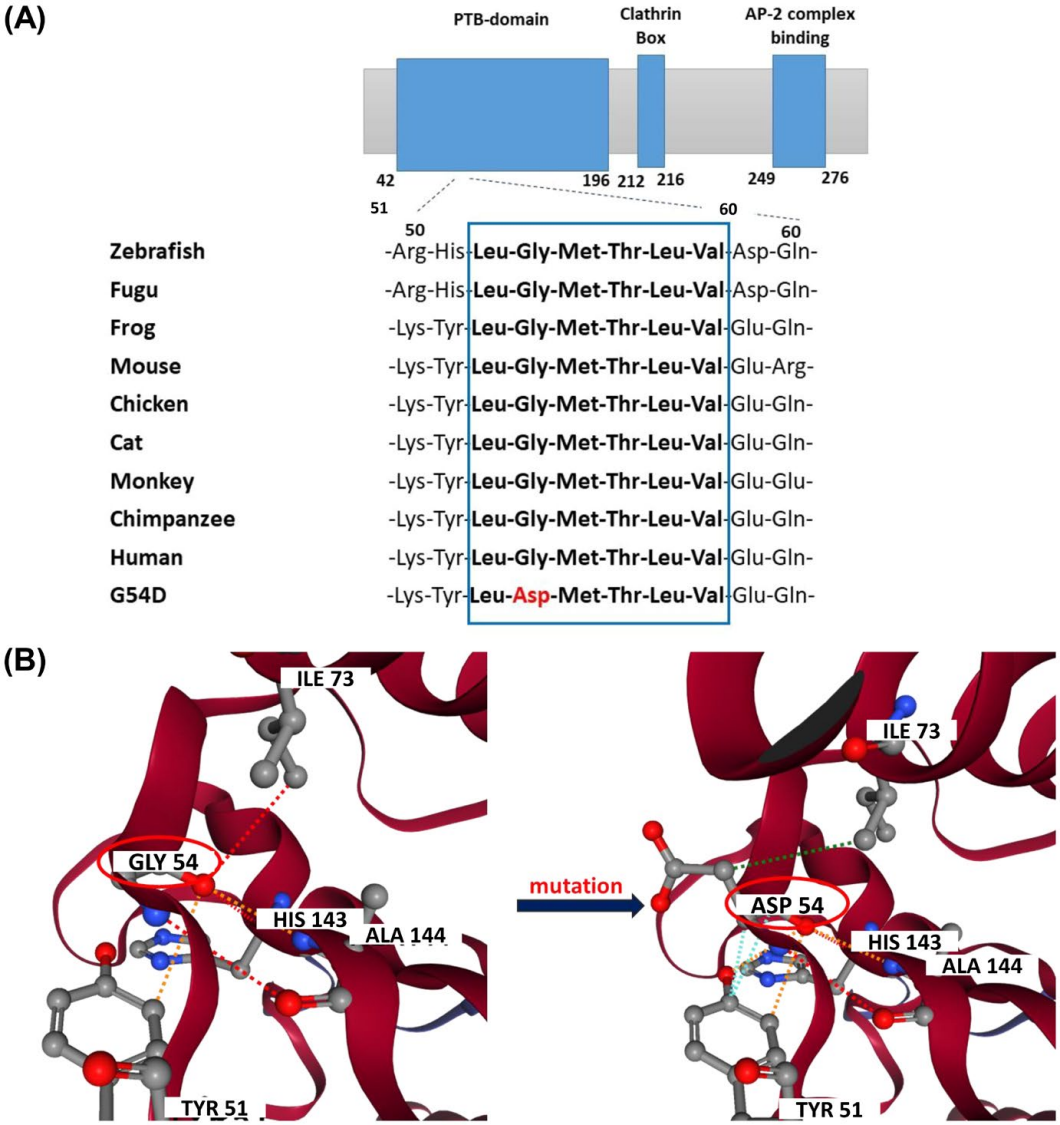
Evolutionary conservation and structural impact of the LDLRAP1 p.Gly54Asp variant. (A) Multiple sequence alignment of LDLRAP1 across nine vertebrate species showing strong evolutionary conservation of Gly54, along with five additional residues highlighted in the blue box. (B) Three‐Dimensional Structural Visualisation of LDLRAP1 Wild‐Type and p.Gly54Asp Variant proteins generated using DynaMut2. Dotted lines indicate molecular interactions: Red for hydrogen bond, orange for polar bond, green for hydrophobic interactions, sky blue for VDW contacts. In the wild‐type protein, Gly54 forms hydrogen bonds with Ile73 and Ala144, stabilising the local structure. In the p.Gly54Asp variant, these hydrogen bonds are lost. The substitution Asp54 instead forms a hydrogen bond with His143, a hydrophobic interaction with Ile73, and a Van der Waals contact with Tyr51. These changes in intra‐protein interactions introduce steric hindrance, potentially disrupting proper protein folding and compromising LDLRAP1 function.

Multiple sequence alignment of LDLRAP1 with nine orthologs revealed that Gly54 is fully conserved across a wide range of mammalian and non‐mammalian species (Figure 4A).

### 3.4 *In Silico* Structural and Functional Characterisation of the LDLRAP1 p.Gly54Asp Variant

The potential structural and functional impact of the novel *LDLRAP1* c.161G>A (p.Gly54Asp) variant was assessed using multiple *in silico* prediction tools, including *MutationTaster, Meta‐SNP, MutPred2* and *CADD*. Given the crucial role of LDLRAP1 in mediating LDL uptake by hepatocytes, computational evaluation of this variant is essential. Predictive analyses consistently indicated a deleterious effect: *MutationTaster* classified the variant as likely pathogenic, supported by a PhastCons score of 1 and PhyloP values of 5.908 and 1.503, reflecting strong evolutionary conservation. *MutPred2* assigned a high pathogenicity score (0.940; *p* = 0.04) and predicted a gain of acetylation at lys51 and potential disruptions in functional motifs listed in the ELM (Eukaryotic Linear Motif) database (e.g., ELME000064, ELME000106, ELME000120), suggesting interference with critical interaction sites. Additionally, the variant obtained a *CADD* score of 32 (chr1:25,553,994; GRCh38‐v1.7), ranking among the top 0.1% most deleterious variants in the human genome, highlighting its potential functional and clinical impact.

Protein stability was evaluated using three complementary computational tools. *DynaMut2*, *MUpro* and *DDGun*. Both *DDGun* and *DynaMut2* predicted destabilising effect on the tertiary structure of LDLRAP1 with a ΔΔG values of −2.5 and −1.56 kcal/mol, respectively. *MUpro* corroborated this conclusion with a ΔΔG of −0.95 kcal/mol, suggesting that the p.Gly54Asp substitution destabilises the protein structure.

Molecular modelling and three‐dimensional visualisation using *DynaMut2* (Figure 4B) revealed significant alterations in the local intra‐protein interactions caused by the p.Gly54Asp variant. In the wild‐type protein, Gly54 forms stabilising hydrogen bonds with Ile73 and Ala144. In contrast, the Asp54 mutant establishes a new hydrogen bond with His143, a hydrophobic interaction with Ile73, and a van der waals contact with Tyr51, leading to a conformational rearrangement likely to impair proper folding and function. The Blosum substitution score for Gly → Asp (−8.563) indicates a highly non‐conservative change that is rarely tolerated in evolution. Additionally, the Kyte‐Doolittle hypopathy score (0.328) suggests that the introduction of a polar aspartate residue in a mildly hydrophobic environment is structurally unfavourable. Comparative sequence analysis across species further supports this interpretation: Gly54 is conserved in 86.4% of orthologous sequences, whereas Asp54 occurs in only 0.5%, highlighting its rarity and likely pathogenicity.

**FIGURE 5:**
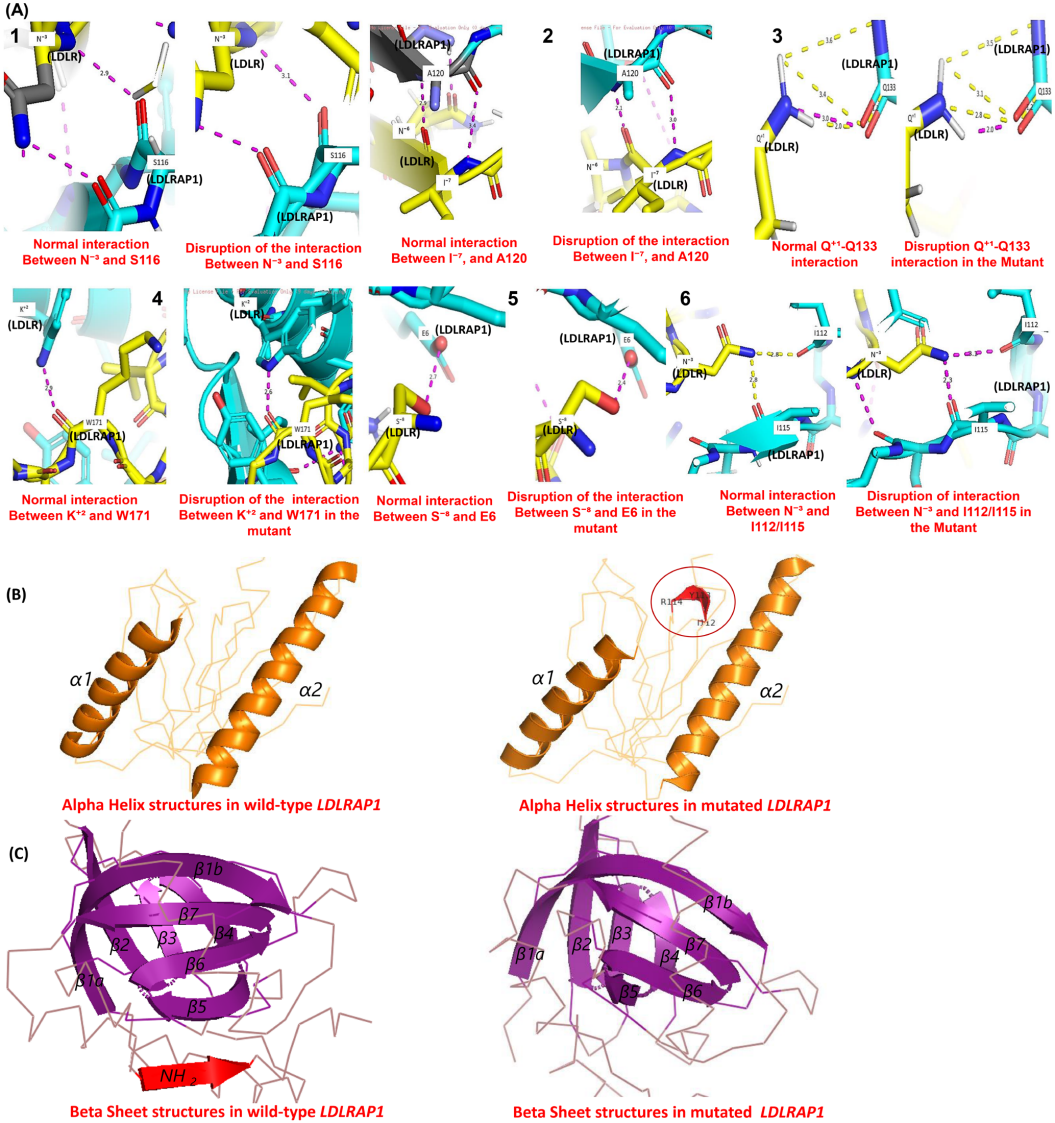
Structural impact of the p.Gly54Asp mutation on LDLRAP1 binding to the LDLR FxNPxY^0^ motif. (A) Structural modelling of the LDLRAP1–LDLR complex shows that in the wild‐type form, multiple hydrogen bonds and polar interactions stabilise the association between LDLRAP1 (light blue) and the *LDLR* FxNPxY^0^ motif (yellow). Key interactions include: (1) Ser116‐Asn^−3^, (2) Ala120 Asn^−6^/Ile^−7^, (3) Gln133‐Gln^+1^, (4) Trp171‐Lys^+2^, (5) Glu6‐Ser^−8^ and (6) Ile115/Ile112‐Asn^−3^. In the p.Gly54Asp variant, several of these contacts are weakened, shortened or reoriented, leading to a loss of stabilising interactions and suggesting impaired *LDLR* internalisation. (B) Secondary structure analysis reveals that wild‐type LDLRAP1 contains two well‐defined α‐helices (α1 and α2), whereas the p.Gly54Asp mutant displays an additional α‐helix formed by residues Ile112, Tyr113 and Arg114 (highlighted in red), indicating altered local folding and potential changes in flexibility. (C) Comparative modelling further shows that wild‐type LDLRAP1 contains eight β‐strands. Upon *LDLR* binding, an additional N‐terminal β‐strand from the LDLR tail aligns with LDLRAP1's β5 strand to form an extended β‐sheet. This β‐strand interaction is absent in the p.Gly54Asp mutant, suggesting reduced structural integrity and weakened receptor binding. Overall, these conformational alterations likely decrease the binding affinity between LDLRAP1 and LDLR, thereby compromising receptor‐mediated endocytosis.

Further structural analysis using *PyMOL* demonstrated that the p.Gly54Asp substitution induces a significant reorganisation of the LDLRAP1–LDLR interface. In the wild‐type complex, a network of hydrogen bonds and polar interactions—(1) Ser116 with Asn^−3^, (2) Ala120 with Asn^−6^ and Ile^−7^, (3) Gln133 with Gln^+1^, (4) Trp171 with Lys^+2^, (5) Glu6 with Ser^−8^ and (6) Ile115 and Ile112 with Asn^−3^—anchors LDLRAP1 to the FxNPxY^0^ motif of LDLR. In the p.Gly54Asp mutant, these stabilising interactions are disrupted: some are weakened or shortened, while others are reoriented or more distant (Figure 5A), resulting in a reconfigured binding surface likely less effective at mediating receptor internalisation.

Secondary structure comparison revealed conformational rearrangements, with the wild‐type LDLRAP1 comprising two α‐helices and eight β‐strands, whereas the mutant adopts three α‐helices and seven β‐strands (Figure 5B,C), suggesting local misfolding that could impair endocytic function. *NetSurfP‐2.0* analysis indicated that the Gly54 is located within a β‐sheet, exposed to solvent at a relative solvent accessibility (RSA) of 18%, and lies in a structurally ordered region, making it a conformationally constrained and functionally significant site. *AlphaFold* predicted this region with high confidence (pLDDT > 90), and *AlphaMissense* classified the variant as probably pathogenic, reinforcing the predicted structural and functional disruption.

Evolutionary conservation analysis using *Consurf* revealed that G54 is highly conserved across species, present in 99% of aligned orthologous sequences (Profile F[G] = 99%, F[D] = 0%), underscoring its critical functional importance. Alterations at such conserved positions are often deleterious. Finally, the meta‐predictor *Meta*‐*SNP*, which integrates *PANTHER*, *PhD*‐*SNP*, *SNAP* and *SIFT* consistently classified the Gly54Asp variant as disease‐associated (PANTHER (0.825), PhD‐SNP (0.865), and SNAP (0.842) and a SIFT score of 0.010), with a Reliability Index of 7/10 and conservation score 0.774.

Collectively, these convergent *in silico* results demonstrate that the *LDLRAP1* p.Gly54Asp variant destabilises protein structure, alters LDLR binding, and likely impairs receptor‐mediated endocytosis, *supporting* its classification as ‘likely pathogenic’ under ACMG criteria.

## 4 Discussion

In this study, we investigated the genetic causes of the familial hypercholesterolemia (FH) in two Tunisian families next‐generation sequencing (NGS) technology, specifically whole‐exome sequencing (WES). In the first family (Family A), we identified a previously reported splice‐site mutation, c.1845+1G>A, in the *LDLR* gene (19p13.2). This variant was present in both affected parents and one daughter in the heterozygous state, and in another daughter in the homozygous state, indicating an autosomal dominant pattern of inheritance (ADH) with one homozygous case. In the second family (Family B), we identified a novel homozygous mutation, c.161G>A (p.Gly54Asp), in the *LDLRAP1* gene (1p36.11) in two affected brothers. According to the ACMG guidelines, the *LDLRAP1* c.161G>A (p.Gly54Asp) variant meets criteria PM1, PM2, PP1 and PP3, and is therefore classified as likely pathogenic. Consistent with this classification, multiple *in silico* prediction tools also support a deleterious effect on protein structure and function [30].

The c.1845+1G>A splice variant, located at the highly conserved GT dinucleotide of the 5′ donor site in intron 12 of *LDLR*, has been previously reported in a patient from New Zealand and in two Tunisian families [27, 38] (Table 1). This variant disrupts normal mRNA splicing, resulting aberrant transcripts and reducing LDL receptor activity to < 2% in homozygous individuals. Functionally, the mutation affects the second domain of the *LDLR*, homologous to the epidermal growth factor (EGF) precursor. Although this domain is not essential for receptor transport or maturation, its disruption impairs acid‐dependent lipoprotein dissociation in endosomes, blocks receptor recycling, and alters the conformational structure required for efficient apoB binding [27].

Plasma lipid levels, expressed as multiples of the median (*MoM*), showed that the homozygous patient IV‐3 had a total cholesterol *MoM* of 6.31 and an LDL‐C *MoM* of 8.24, over threefold higher than previously reported in Jelassi et al. homozygous patients [27]. MoM values were calculated based on the lipid levels presented in Table 2. This discrepancy could be attributed to several factors, including diet, lifestyle, treatment adherence and genetic background. In our study, all patients from Family A were treated with statin (*atorvastatin* and *rosuvastatin*), and the father (III‐10) who present cardiovascular complications receives an additional cholesterol‐lowering agent that reduces intestinal cholesterol absorption (*ezetimibe*). No information regarding the treatment was reported in the study of Jelassi et al.

A recent meta‐analysis indicated that, although statins are commonly prescribed, individual responses vary markedly (10%–50% differences in efficacy and toxicity), influenced not only by clinical factors but also by genetic polymorphisms. In particular, the meta‐analysis highlighted that polymorphisms in *SLCO1B1* play a major role in modulating the response to statin therapy. The study also mentioned other genetic variants, including *APOE*, *LPA*, *SORT1* and *ABCG2*, which have been reported in the literature as potentially influencing statin effectiveness [39, 40]. None of these polymorphisms were identified in our patients. Jelassi et al. sequenced only *LDLR* and *PCSK9*, without broader next‐generation sequencing (NGS), which may partly explain the limited genetic insights. This limitation is further illustrated by the identification of two *PCSK9* variants (p.Phe515Leu and p.Gly670Glu (*rs505151*)) in addition to the mutation in *LDLR*.

The p.Phe515Leu variant, first reported by Kotowski et al., it is extremely rare and initially classified as a variant of uncertain significance. It affects a highly conserved residue in the C‐terminal domain of *PCSK9*. Functional and structural analyses, particularly by Sarkar et al., showed that this variant reduces *PCSK9's* binding to LDL particles, enhancing LDL receptor degradation and producing an autosomal dominant FH phenotype [41, 42].

The *rs505151* variant is generally considered a gain‐of‐function pathogenic variant that promotes LDLR degradation and increases plasma LDL‐C levels. Carriers of the G allele in Tunisian, Chinese and European cohorts, exhibited elevated LDL‐C, polygenic hypercholesterolemia in men, and more severe coronary atherosclerosis [43, 44, 45, 46]. In Jelassi et al.'s study, all homozygotes presented skin xanthomas, with 75% showing coronary heart diseases, whereas heterozygotes were largely unaffected [27]. In contrast, no significant association with coronary artery disease was reported in Taiwanese and Indian studies and in some cases, carriers even displayed lower LDL‐C levels [47, 48]. These observations collectively suggest that lipid profiles and clinical outcomes are multifactorial, shaped by genetic background, treatment, and environmental influences, highlighting the important considering both variant‐specific and individual context in interpreting phenotypes.

Variants in the *LDLRAP1* gene, which are transmitted in an autosomal recessive manner, have not previously been reported in Tunisian patients with FH. To date, 158 ARH patients have been described worldwide, mostly from Sardinia (Italy), suggesting a founder effect. Other cases have been reported in Spain, United States, Japan, Iran, India, United Kingdom, Turkey, Mexico, Syria and Lebanon [49, 50, 51, 52, 53, 54, 55, 56, 57, 58, 59, 60, 61, 62, 63, 64, 65, 66, 67, 68].

According to the Varsome database (https://varsome.com/gene/hg38/LDLRAP1), 454 clinical variants have been reported in the *LDLRAP1*. Among these, 71 are classified as pathogenic, including only six missense mutations. Additionally, 173 variants are of uncertain significance, and 216 are classified as benign. The most frequent pathogenic variant is: chr1:g.25,563,142C>A (GRCh38) (c.605C>A, p.S202Y, NM_015627.3) with an African frequency = 0.003176; https://varsome.com/gene/hg38/LDLRAP1.

The missense variant c.161G>A, (p.Gly54Asp) identified in our study lies within the PTB domain, which forms part of the lipid‐binding adaptor protein involved in multiple physiological processes, including binding to the NPXY internalisation signal of the LDLR [69].

In ARH patients, LDLR function is severely impaired in hepatocytes, despite increased receptor expression on lymphocytes, which does not result in effective LDL uptake. Fibroblasts retain partial LDL internalisation and degradation (≈70%–75% and 60%–65% of normal), distinguishing ARH from HoFH [70]. ARH lymphocytes show high surface LDLR but reduced LDL uptake, whereas LDLR‐mutant cells display more severe impairment [8]. Nevertheless, ARH cells partly downregulate LDLR in response to extracellular LDL, indicating that LDLRAP1 is essential but not the sole regulator of LDL internalisation [71]. Restoration of wild‐type LDLRAP1 cDNA rescues internalisation, confirming its critical role [72].

The *LDLRAP1* variant c.161G>A (p.Gly54Asp) was classified as likely pathogenic based on its rarity, homozygous state, location within a critical functional domain, co‐segregation with affected family members, and supportive *in silico* prediction.

Clinically, while heterozygous parents were completely asymptomatic, the two ARH patients presented yellowish xanthomas from age 4 on knees, tendons and elbows, including a large xanthoma on the left elbow (Figure 2). LDL‐C levels were markedly elevated (5.75 and 6.90 mmol/L). These patients also exhibited a quadricuspid aortic valve (QAV); although no direct link with hypercholesterolemia has been established, it has been suggested that elevated cholesterol may contribute to valvular disease [73, 74, 75]. Screening of major valvulogenesis genes (*NKX2*‐*5*, *TBX5*, *GATA4*, *MMP2*) revealed no co‐segregating pathogenic variants in these ARH patients.

Variants in other valvulogenesis genes including *NOTCH1*, *AGTR1*, *NKX2*‐6 and *BMP2* were identified in the two patients and their mother. However, their contribution remains uncertain due to lack of echocardiographic confirmation and absence of validation by Sanger sequencing. A co‐segregating synonymous variant in *MMP2* was also identified. Although this variant does not alter the protein sequence, it may influence gene expression through epigenetic mechanisms, such as DNA methylation, microRNA regulation, or mRNA translation efficiency. Supporting this hypothesis, Martin et al. reported several synonymous variants co‐segregating in extended families, consistent with the inheritance patterns observed in bicuspid aortic valve (BAV) [76, 77].

It is also important to note that QAV is an extremely rare congenital anomaly in FH, with only one case previously associated with a *SALL4* variant [78, 79]. Excess cholesterol during embryogenesis can disrupt Notch1 signalling, impairing valve morphogenesis through abnormal differentiation of endothelial and fibroblast cells, oxidative stress, and premature calcification. In the context of LDLRAP1 deficiency, intrauterine LDL accumulation may thus create an adverse developmental environment, indirectly affecting valve formation, highlighting the need for further research.

Collectively, these findings indicate that genetic testing is essential for the accurate diagnosis of familial hypercholesterolemia (FH), particularly in atypical cases, as it enables precise subtype classification, cascade screening, and timely intervention. By distinguishing heterozygous from homozygous or compound heterozygous patients, genetic analysis directly informs prognosis and therapeutic decisions, supporting personalised management strategies. This is especially important for rare forms such as ARH, as highlighted in our study. Early diagnosis, particularly in children, opens a critical window for preventive lifestyle measures and pharmacological therapy, including early statin initiation. Overall, integrating genetic screening with individualised treatment plans is key to reducing cardiovascular morbidity and mortality in FH, while empowering families and clinicians to implement proactive and personalised measures for cardiovascular risk prevention.

## 5 Conclusion

This study provides the first molecular evidence in Tunisia linking pathogenic variants in *LDLR* (c.1845+1G>A) and *LDLRAP1* (c.161G>A; p.Gly54Asp) to familial hypercholesterolemia (FH). These findings expand the mutational spectrum of FH in North Africa and illustrate the coexistence of both autosomal dominant and recessive inheritance patterns underlying severe hypercholesterolemia. The identified variants explain the pronounced clinical phenotype observed in affected individuals and underscore the importance of integrating comprehensive genetic testing into FH diagnosis. Early molecular screening can facilitate accurate risk stratification and family counselling, while supporting precision‐based therapeutic strategies to prevent premature cardiovascular disease.

## Author Contributions

W.B.N. conducted comprehensive data and bioinformatics analysis and contributed to the writing of the original manuscript. A.B.‐M. performed exome sequencing of the patients, data filtration, analysis and interpretation. H.F. oversaw the accurate collection of DNA samples and associated clinical information. F.H.K. and N.M. played a key role in patient recruitment and the collection of clinical data. H.‐G.K. critically reviewed, revised, and finalised the manuscript, providing scientific guidance and oversight throughout the study. M.M. contributed to patient recruitment and coordination of field data collection. J.‐J.H. revised the manuscript and providing scientific guidance throughout the study. L.A.K. coordinated the overall planning of the project and actively participated in drafting and structuring the manuscript. F.A. participated in drafting and structuring the manuscript.

## Funding

The laboratory of J.‐J.H. is funded by internal grant QB35 at by Qatar Biomedical Research Institute, Hamad Bin Khlifa University. This work was supported by the Laboratory of Human Molecular Genetics at the Faculty of Medicine of Sfax, and the Endocrinology Department of Hedi Chaker University Hospital, Sfax.

## Disclosure

Web resources: gnomAD, https://gnomad.broadinstitute.org/; Varsome, https://varsome.com/; SIFT, https://sift.bii.a‐star.edu.sg/; PolyPhen‐2, https://bio.tools/polyphen‐2; ClinVar, https://www.ncbi.nlm.nih.gov/clinvar/; UniProt, https://www.uniprot.org/; RCSB PDB, https://www.rcsb.org/; Ensembl, https://www.ensembl.org/index.html; DynaMut2, https://biosig.lab.uq.edu.au/dynamut2/; MUpro, https://mupro.proteomics.ics.uci.edu/; DDGun, https://folding.biofold.org/ddgun/; NetSurfP‐2.0, https://services.healthtech.dtu.dk/services/NetSurfP‐2.0/; AlphaFold, https://alphafold.ebi.ac.uk/; Consurf, https://consurf.tau.ac.il/consurf_index.php; MutationTaster, https://www.mutationtaster.org/; Meta‐SNP, https://snps.biofold.org/meta‐snp/; CADD, https://cadd.gs.washington.edu/; MutPred2, https://mutpred.mutdb.org.

## Consent

Informed consent for the publication of the anonymized data was obtained from all participants or their legal guardians, in accordance with ethical guidelines and the study protocol approved by the institutional review board of the Faculty of Medicine of Sfax.

## Conflicts of Interest

The authors declare no conflicts of interest.

## Supporting information


**Table S1:** Primer sequences and corresponding annealing temperature.

## Data Availability

The data that support the findings of this study are available from the corresponding author upon reasonable request.

## References

[1] B. A. Ference , H. N. Ginsberg , I. Graham , et al., “Low‐Density Lipoproteins Cause Atherosclerotic Cardiovascular Disease. 1. Evidence From Genetic, Epidemiologic, and Clinical Studies. A Consensus Statement From the European Atherosclerosis Society Consensus Panel,” European Heart Journal 38, no. 32 (2017): 2459–2472. 28444290 10.1093/eurheartj/ehx144 PMC5837225

[2] P. Hu , K. I. Dharmayat , C. A. T. Stevens , and R. S. Jones , “Prevalence of Familial Hypercholesterolemia Among the General Population and Patients With Atherosclerotic Cardiovascular Disease: A Systematic Review and Meta‐Analysis,” (2020), accessed September 21, 2025, https://pubmed.ncbi.nlm.nih.gov/32468833/. 10.1161/CIRCULATIONAHA.119.044795 32468833

[3] J. P. S. Sawhney and K. Madan , “Familial Hypercholesterolemia,” Indian Heart Journal 76 Suppl 1, no. Suppl 1 (2024): S108–S112. 38599725 10.1016/j.ihj.2023.12.002 PMC11019323

[4] L. D'Erasmo , A. Di Costanzo , and M. Arca , “Autosomal Recessive Hypercholesterolemia: Update for 2020,” Current Opinion in Lipidology 31, no. 2 (2020): 56–61. 32011344 10.1097/MOL.0000000000000664

[5] R. Henderson , M. O'Kane , and V. McGilligan , “The Genetics and Screening of Familial Hypercholesterolaemia,” (2016), accessed September 21, 2025, https://pubmed.ncbi.nlm.nih.gov/27084339/. 10.1186/s12929-016-0256-1 PMC4833930 27084339

[6] N. Ahangari , A. Sahebkar , M. Azimi‐Nezhad , et al., “A Novel Splice Site Variant in the LDLRAP1 Gene Causes Familial Hypercholesterolemia,” Iranian Biomedical Journal 25, no. 5 (2021): 374–379. 34425670 10.52547/ibj.25.5.374 PMC8487678

[7] A. J. Berberich and R. A. Hegele , “The Complex Molecular Genetics of Familial Hypercholesterolaemia,” Nature Reviews. Cardiology 16, no. 1 (2019): 9–20. 29973710 10.1038/s41569-018-0052-6

[8] C. Rodríguez‐Jiménez , D. Gómez‐Coronado , M. Frías Vargas , et al., “A New Variant (c.1A>G) in LDLRAP1 Causing Autosomal Recessive Hypercholesterolemia: Characterization of the Defect and Response to PCSK9 Inhibition,” Atherosclerosis 284 (2019): 223–229. 30777337 10.1016/j.atherosclerosis.2019.01.010

[9] P. Nikasa , T. Tricot , N. Mahdieh , et al., “Patient‐Specific Induced Pluripotent Stem Cell‐Derived Hepatocyte‐Like Cells as a Model to Study Autosomal Recessive Hypercholesterolemia,” Stem Cells and Development 30, no. 14 (2021): 714–724. 33938231 10.1089/scd.2020.0199

[10] M. Abifadel and C. Boileau , “Genetic and Molecular Architecture of Familial Hypercholesterolemia,” (2022), accessed September 21, 2025, https://pubmed.ncbi.nlm.nih.gov/36196022/. 10.1111/joim.13577 PMC10092380 36196022

[11] Y. A. Khalil , J. P. Rabès , C. Boileau , and M. Varret , “APOE Gene Variants in Primary Dyslipidemia,” Atherosclerosis 328 (2021): 11–22. 34058468 10.1016/j.atherosclerosis.2021.05.007

[12] F. Civeira , “APOE and Familial Hypercholesterolemia,” (2024), accessed September 21, 2025, https://pubmed.ncbi.nlm.nih.gov/38640077/. 10.1097/MOL.0000000000000937 38640077

[13] S. E. van den Bosch , B. A. Hutten , W. E. Corpeleijn , and D. M. Kusters , “Familial Hypercholesterolemia in Children and the Importance of Early Treatment,” Current Opinion in Lipidology 35, no. 3 (2024): 126–132. 38363694 10.1097/MOL.0000000000000926 PMC11188623

[14] M. Klevmoen , J. W. C. M. Mulder , J. E. van Roeters Lennep , and K. B. Holven , “Sex Differences in Familial Hypercholesterolemia,” Current Atherosclerosis Reports 25, no. 11 (2023): 861–868. 37815650 10.1007/s11883-023-01155-6 PMC10618303

[15] S. D. De Ferranti , A. M. Rodday , M. M. Mendelson , J. B. Wong , L. K. Leslie , and R. C. Sheldrick , “Prevalence of Familial Hypercholesterolemia in the 1999 to 2012 United States National Health and Nutrition Examination Surveys (NHANES),” Circulation 133, no. 11 (2016): 1067–1072. 26976914 10.1161/CIRCULATIONAHA.115.018791

[16] F. Toft‐Nielsen , F. Emanuelsson , and M. Benn , “Familial Hypercholesterolemia Prevalence Among Ethnicities—Systematic Review and Meta‐Analysis,” Frontiers in Genetics 13 (2022): 840797. 35186049 10.3389/fgene.2022.840797 PMC8850281

[17] M. K. Ito and G. F. Watts , “Challenges in the Diagnosis and Treatment of Homozygous Familial Hypercholesterolemia,” Drugs 75, no. 15 (2015): 1715–1724. 26370207 10.1007/s40265-015-0466-y PMC4611011

[18] R. Luo , F. Qingan , J. Wang , L. Hu , S. Zhang , and J. Long , “Frontiers | Applications of Machine Learning in Familial Hypercholesterolemia,” (2023), accessed December 20, 2024, https://www.frontiersin.org/journals/cardiovascular‐medicine/articles/10.3389/fcvm.2023.1237258/full. 10.3389/fcvm.2023.1237258 PMC10562581 37823179

[19] P. Lázaro , L. Pérez de Isla , G. F. Watts , et al., “Cost‐Effectiveness of a Cascade Screening Program for the Early Detection of Familial Hypercholesterolemia,” Journal of Clinical Lipidology 11, no. 1 (2017): 260–271. 28391894 10.1016/j.jacl.2017.01.002

[20] “Limited Mutational Heterogeneity in the LDLR Gene in Familial Hypercholesterolemia in Tunisia,” accessed July 4, 2025, https://pubmed.ncbi.nlm.nih.gov/18757057/. 10.1016/j.atherosclerosis.2008.07.011 18757057

[21] A. Jelassi , A. Slimani , I. Jguirim , et al., “Moderate Phenotypic Expression of Familial Hypercholesterolemia in Tunisia,” Clinica Chimica Acta 411, no. 9–10 (2010): 735–738. 10.1016/j.cca.2010.02.008 20144596

[22] A. Jelassi , A. Slimani , J. P. Rabès , et al., “Genomic Characterization of Two Deletions in the LDLR Gene in Tunisian Patients With Familial Hypercholesterolemia,” Clinica Chimica Acta 414 (2012): 146–151. 10.1016/j.cca.2012.08.002 22910581

[23] A. Jelassi , I. Jguirim , M. Najah , et al., “Limited Mutational Heterogeneity in the LDLR Gene in Familial Hypercholesterolemia in Tunisia,” Atherosclerosis 203, no. 2 (2009): 449–453. 18757057 10.1016/j.atherosclerosis.2008.07.011

[24] M. N. Slimane , S. Lestavel , V. Clavey , et al., “CYS127S (FH‐Kairouan) and D245N (FH‐Tozeur) Mutations in the LDL Receptor Gene in Tunisian Families With Familial Hypercholesterolaemia,” Journal of Medical Genetics 39, no. 11 (2002): e74. 12414836 10.1136/jmg.39.11.e74 PMC1735002

[25] A. Jelassi , M. Najah , I. Jguirim , et al., “A Novel Splice Site Mutation of the LDL Receptor Gene in a Tunisian Hypercholesterolemic Family,” Clinica Chimica Acta 392, no. 1–2 (2008): 25–29. 10.1016/j.cca.2008.02.019 18355452

[26] M. N. Slimane , S. Lestavel , X. Sun , et al., “Fh‐Souassi: A Founder Frameshift Mutation in Exon 10 of the LDL‐Receptor Gene, Associated With a Mild Phenotype in Tunisian Families,” Atherosclerosis 154, no. 3 (2001): 557–565. 11257256 10.1016/s0021-9150(00)00572-4

[27] A. Jelassi , A. Slimani , I. Jguirim , et al., “Effect of a Splice Site Mutation in LDLR Gene and Two Variations in PCSK9 Gene in Tunisian Families With Familial Hypercholesterolaemia,” Annals of Clinical Biochemistry 48, no. Pt 1 (2011): 83–86. 21115573 10.1258/acb.2010.010087

[28] A. Slimani , A. Jelassi , I. Jguirim , et al., “Effect of Mutations in LDLR and PCSK9 Genes on Phenotypic Variability in Tunisian Familial Hypercholesterolemia Patients,” Atherosclerosis 222, no. 1 (2012): 158–166. 22417841 10.1016/j.atherosclerosis.2012.02.018

[29] V. Gupta , A. Ben‐Mahmoud , B. Ku , et al., “Identification of Two Novel Autism Genes, TRPC4 and SCFD2, in Qatar Simplex Families Through Exome Sequencing,” Frontiers in Psychiatry 14 (2023): 1251884. 38025430 10.3389/fpsyt.2023.1251884 PMC10644705

[30] S. Richards , N. Aziz , S. Bale , et al., “Standards and Guidelines for the Interpretation of Sequence Variants: A Joint Consensus Recommendation of the American College of Medical Genetics and Genomics and the Association for Molecular Pathology,” Genetics in Medicine 17, no. 5 (2015): 405–424. 25741868 10.1038/gim.2015.30 PMC4544753

[31] L. Montanucci , E. Capriotti , Y. Frank , N. Ben‐Tal , and P. Fariselli , “DDGun: An Untrained Method for the Prediction of Protein Stability Changes Upon Single and Multiple Point Variations,” BMC Bioinformatics 20, no. Suppl 14 (2019): 335. 31266447 10.1186/s12859-019-2923-1 PMC6606456

[32] P. Corrado , B. Silivia , B. Giovanni , A. Virginia , and R. Valeria , “Predicting Protein Stability Changes Upon Single‐Point Mutation: A Thorough Comparison of the Available Tools on a New Dataset,” (2022), accessed December 20, 2024, https://pubmed.ncbi.nlm.nih.gov/35021190/. 10.1093/bib/bbab555 PMC8921618 35021190

[33] H. M. R. Carlos , E. V. P. Douglas , and B. A. David , “DynaMut2: Assessing Changes in Stability and Flexibility Upon Single and Multiple Point Missense Mutations—Rodrigues—2021—Protein Science,” (2020), accessed December 20, 2024, 10.1002/pro.3942. PMC7737773 32881105

[34] M. S. Klausen , M. C. Jespersen , H. Nielsen , et al., “NetSurfP‐2.0: Improved Prediction of Protein Structural Features by Integrated Deep Learning,” Proteins: Structure, Function, and Bioinformatics 87, no. 6 (2019): 520–527. 10.1002/prot.25674 30785653

[35] Y. Zhenyu , Z. Xiaoxi , Z. Yi , and C. Runsheng , “AlphaFold2 and Its Applications in the Fields of Biology and Medicine,” (2023), accessed December 21, 2024, https://pubmed.ncbi.nlm.nih.gov/36918529/.

[36] H. Ashkenazy , S. Abadi , E. Martz , et al., “ConSurf 2016: An Improved Methodology to Estimate and Visualize Evolutionary Conservation in Macromolecules,” Nucleic Acids Research 44, no. Web Server issue (2016): W344–W350. 27166375 10.1093/nar/gkw408 PMC4987940

[37] S. Rosignoli and A. Paiardini , “Boosting the Full Potential of PyMOL With Structural Biology Plugins,” Biomolecules 12, no. 12 (2022): 1764. 36551192 10.3390/biom12121764 PMC9775141

[38] A. D. Laurie , R. S. Scott , and P. M. George , “Genetic Screening of Patients With Familial Hypercholesterolaemia (FH): A New Zealand Perspective,” Atherosclerosis. Supplements 5, no. 5 (2004): 13–15. 15556094 10.1016/j.atherosclerosissup.2004.09.001

[39] H. H. Nguyen , C. T. T. Nguyen , T. N. P. Mai , and P. T. Huong , “Associations Between Four Polymorphisms of the SLCO1B1 and Effectiveness of the Statins: A Meta‐Analysis,” Pharmacogenetics and Genomics 33, no. 4 (2023): 65–78. 37098851 10.1097/FPC.0000000000000490

[40] M. Leusink , N. C. Onland‐Moret , P. I. W. de Bakker , A. de Boer , and A. H. van der Maitland‐Zee , “Seventeen Years of Statin Pharmacogenetics: A Systematic Review,” Pharmacogenomics 17, no. 2 (2016): 163–180. 26670324 10.2217/pgs.15.158

[41] I. K. Kotowski , A. Pertsemlidis , A. Luke , et al., “A Spectrum of PCSK9 Alleles Contributes to Plasma Levels of Low‐Density Lipoprotein Cholesterol,” American Journal of Human Genetics 78, no. 3 (2006): 410–422. 16465619 10.1086/500615 PMC1380285

[42] S. K. Sarkar , A. C. Y. Foo , A. Matyas , et al., “A Transient Amphipathic Helix in the Prodomain of PCSK9 Facilitates Binding to Low‐Density Lipoprotein Particles,” Journal of Biological Chemistry 295, no. 8 (2020): 2285–2298. 31949048 10.1074/jbc.RA119.010221 PMC7039556

[43] A. Slimani , Y. Harira , I. Trabelsi , et al., “Effect of E670G Polymorphism in PCSK9 Gene on the Risk and Severity of Coronary Heart Disease and Ischemic Stroke in a Tunisian Cohort,” Journal of Molecular Neuroscience 53, no. 2 (2014): 150–157. 24599757 10.1007/s12031-014-0238-2

[44] D. Evans and F. U. Beil , “The E670G SNP in the PCSK9 Gene Is Associated With Polygenic Hypercholesterolemia in Men but Not in Women,” BMC Medical Genetics 7 (2006): 66. 16875509 10.1186/1471-2350-7-66 PMC1562364

[45] “A Common PCSK9 Haplotype, Encompassing the E670G Coding Single Nucleotide Polymorphism, Is a Novel Genetic Marker for Plasma Low‐Density Lipoprotein Cholesterol Levels and Severity of Coronary Atherosclerosis,” accessed October 7, 2025, https://pubmed.ncbi.nlm.nih.gov/15893176/. 10.1016/j.jacc.2005.01.051 PMC2910256 15893176

[46] X. M. He , L. Chen , T. S. Wang , Y. B. Zhang , J. B. Luo , and X. X. Feng , “E670G Polymorphism of PCSK9 Gene of Patients With Coronary Heart Disease Among Han Population in Hainan and Three Provinces in the Northeast of China,” Asian Pacific Journal of Tropical Medicine 9, no. 2 (2016): 172–176. 26919950 10.1016/j.apjtm.2016.01.008

[47] S. M. Chiang , Y. S. Yang , S. F. Yang , C. F. Tsai , and K. C. Ueng , “Variations of the Proprotein Convertase Subtilisin/Kexin Type 9 Gene in Coronary Artery Disease,” Journal of International Medical Research 48, no. 1 (2020): 300060519839519. 30947598 10.1177/0300060519839519 PMC7140201

[48] A. Eba , S. T. Raza , I. A. Wani , et al., “Association of APOB (rs515135) and PCSK9 (rs505151) Gene Polymorphisms With CAD in the Indian Population,” Biomarkers in Medicine 19, no. 10 (2025): 371–377. 40270278 10.1080/17520363.2025.2496128 PMC12077466

[49] J. M. Mostaza and C. Escobar , “Rosuvastatin‐Based Lipid‐Lowering Therapy for the Control of LDL Cholesterol in Patients at High Vascular Risk,” Journal of Clinical Medicine 13, no. 7 (2024): 1894. 38610659 10.3390/jcm13071894 PMC11012264

[50] J. S. Dron , J. Wang , A. D. McIntyre , et al., “Six Years' Experience With LipidSeq: Clinical and Research Learnings From a Hybrid, Targeted Sequencing Panel for Dyslipidemias,” BMC Medical Genomics 13, no. 1 (2020): 23. 32041611 10.1186/s12920-020-0669-2 PMC7011550

[51] E. R. Eden , R. P. Naoumova , J. J. Burden , M. I. McCarthy , and A. K. Soutar , “Use of Homozygosity Mapping to Identify a Region on Chromosome 1 Bearing a Defective Gene That Causes Autosomal Recessive Homozygous Hypercholesterolemia in Two Unrelated Families,” American Journal of Human Genetics 68, no. 3 (2001): 653–660. 11179013 10.1086/318795 PMC1274478

[52] C. M. Barbagallo , G. Emmanuele , A. B. Cefalù , et al., “Autosomal Recessive Hypercholesterolemia in a Sicilian Kindred Harboring the 432insA Mutation of the ARH Gene,” (2003), accessed December 23, 2024, https://pubmed.ncbi.nlm.nih.gov/12535754/. 10.1016/s0021-9150(02)00379-9 12535754

[53] M. Arca , G. Zuliani , K. Wilund , et al., “Autosomal Recessive Hypercholesterolaemia in Sardinia, Italy, and Mutations in ARH: A Clinical and Molecular Genetic Analysis,” Lancet 359, no. 9309 (2002): 841–847. 11897284 10.1016/S0140-6736(02)07955-2

[54] R. Fellin , G. Zuliani , M. Arca , et al., “Clinical and Biochemical Characterisation of Patients With Autosomal Recessive Hypercholesterolemia (ARH),” Nutrition, Metabolism, and Cardiovascular Diseases 13, no. 5 (2003): 278–286. 10.1016/s0939-4753(03)80032-7 14717060

[55] O. Marmontel , P. A. Rollat‐Farnier , A. S. Wozny , et al., “Development of a New Expanded Next‐Generation Sequencing Panel for Genetic Diseases Involved in Dyslipidemia,” Clinical Genetics 98, no. 6 (2020): 589–594. 33111339 10.1111/cge.13832

[56] M. H. Martinsen , I. C. Klausen , A. Tybjaerg‐Hansen , and B. S. Hedegaard , “Autosomal Recessive Hypercholesterolemia in a Kindred of Syrian Ancestry,” Journal of Clinical Lipidology 14, no. 4 (2020): 419–424. 32636080 10.1016/j.jacl.2020.06.002

[57] S. Muntoni , L. Pisciotta , S. Muntoni , and S. Bertolini , “Pharmacological Treatment of a Sardinian Patient Affected by Autosomal Recessive Hypercholesterolemia (ARH),” Journal of Clinical Lipidology 9, no. 1 (2015): 103–106. 25670367 10.1016/j.jacl.2014.08.009

[58] R. Spina , D. Noto , C. M. Barbagallo , et al., “Genetic Epidemiology of Autosomal Recessive Hypercholesterolemia in Sicily: Identification by Next‐Generation Sequencing of a New Kindred,” Journal of Clinical Lipidology 12, no. 1 (2018): 145–151. 29153781 10.1016/j.jacl.2017.10.014

[59] A. C. Sturm , R. Truty , T. E. Callis , et al., “Limited‐Variant Screening vs Comprehensive Genetic Testing for Familial Hypercholesterolemia Diagnosis,” JAMA Cardiology 6, no. 8 (2021): 902–909. 34037665 10.1001/jamacardio.2021.1301 PMC8156154

[60] R. M. Sánchez‐Hernández , P. Prieto‐Matos , F. Civeira , et al., “Autosomal Recessive Hypercholesterolemia in Spain,” Atherosclerosis 269 (2018): 1–5. 29245109 10.1016/j.atherosclerosis.2017.12.006

[61] M. Harada‐Shiba , A. Takagi , Y. Miyamoto , et al., “Clinical Features and Genetic Analysis of Autosomal Recessive Hypercholesterolemia,” Journal of Clinical Endocrinology and Metabolism 88, no. 6 (2003): 2541–2547. 12788851 10.1210/jc.2002-021487

[62] A. Pirillo , K. Garlaschelli , M. Arca , et al., “Spectrum of Mutations in Italian Patients With Familial Hypercholesterolemia: New Results From the LIPIGEN Study,” Atherosclerosis. Supplements 29 (2017): 17–24. 28965616 10.1016/j.atherosclerosissup.2017.07.002

[63] H. Tada , M. Kawashiri , R. Ohtani , et al., “A Novel Type of Familial Hypercholesterolemia: Double Heterozygous Mutations in LDL Receptor and LDL Receptor Adaptor Protein 1 Gene,” Atherosclerosis 219, no. 2 (2011): 663–666. 21872251 10.1016/j.atherosclerosis.2011.08.004

[64] H. Tada , M. Kawashiri , K. Ikewaki , et al., “Altered Metabolism of Low‐Density Lipoprotein and Very‐Low‐Density Lipoprotein Remnant in Autosomal Recessive Hypercholesterolemia,” Circulation: Cardiovascular Genetics 5, no. 1 (2012): 35–41. 22157599 10.1161/CIRCGENETICS.111.960948

[65] C. Q. Samuel , A. A. S. Carlos , H. V. Adriana , L. O. S. María , and R. T. Maribel , “A Novel ARH Splice Site Mutation in a Mexican Kindred With Autosomal Recessive Hypercholesterolemia,” (2005), accessed December 23, 2024, https://pubmed.ncbi.nlm.nih.gov/15599766/. 10.1007/s00439-004-1192-9 15599766

[66] B. Bobrowska , W. Zasada , M. Zawada , A. Surdacki , S. Bartuś , and R. Rajtar‐Salwa , “Autosomal Recessive Hypercholesterolemia: The First Experience in Poland,” Polskie Archiwum Medycyny Wewnętrznej 133, no. 6 (2023): 16498. 37171179 10.20452/pamw.16498

[67] S. L. T. Pek , F. Yap , A. V. Sreedharan , J. T. L. Choo , and S. Tavintharan , “Persistent Hypercholesterolemia in Child With Homozygous Autosomal Recessive Hypercholesterolemia: A Decade of Lipid Management,” Journal of Clinical Lipidology 15, no. 3 (2021): 441–446. 33994332 10.1016/j.jacl.2021.04.008

[68] P. Nikasa , B. Rabbani , M. S. Hejazi , et al., “A Case of Autosomal Recessive Hypercholesterolemia With a Novel Mutation in the LDLRAP1 Gene,” Clinical Pediatric Endocrinology 30, no. 4 (2021): 201–204. 34629743 10.1297/cpe.30.201 PMC8481080

[69] G. He , S. Gupta , M. Yi , P. Michaely , H. H. Hobbs , and J. C. Cohen , “ARH Is a Modular Adaptor Protein That Interacts With the LDL Receptor, Clathrin, and AP‐2,” Journal of Biological Chemistry 277, no. 46 (2002): 44044–44049. 12221107 10.1074/jbc.M208539200

[70] R. Fellin , M. Arca , G. Zuliani , S. Calandra , and S. Bertolini , “The History of Autosomal Recessive Hypercholesterolemia (ARH). From Clinical Observations to Gene Identification,” Gene 555, no. 1 (2015): 23–32. 25225128 10.1016/j.gene.2014.09.020

[71] K. R. Wilund , M. Yi , F. Campagna , et al., “Molecular Mechanisms of Autosomal Recessive Hypercholesterolemia,” Human Molecular Genetics 11, no. 24 (2002): 3019–3030. 12417523 10.1093/hmg/11.24.3019

[72] E. R. Eden , D. D. Patel , X. M. Sun , et al., “Restoration of LDL Receptor Function in Cells From Patients With Autosomal Recessive Hypercholesterolemia by Retroviral Expression of ARH1,” Journal of Clinical Investigation 110, no. 11 (2002): 1695–1702. 12464675 10.1172/JCI16445 PMC151635

[73] S. Saith , S. Saith , and A. Murthy , “Quadricuspid Aortic Valve: An Introduction for Clinicians,” Cardiology Research 13, no. 1 (2022): 2–10. 35211218 10.14740/cr1308 PMC8827235

[74] J. G. Smith , K. Luk , C. A. Schulz , et al., “Association of Low‐Density Lipoprotein Cholesterol‐Related Genetic Variants With Aortic Valve Calcium and Incident Aortic Stenosis,” Journal of the American Medical Association 312, no. 17 (2014): 1764–1771. 25344734 10.1001/jama.2014.13959 PMC4280258

[75] N. M. Rajamannan , W. D. Edwards , and T. C. Spelsberg , “Hypercholesterolemic Aortic‐Valve Disease,” New England Journal of Medicine 349, no. 7 (2003): 717–718. 12917318 10.1056/NEJMc031360 PMC3951872

[76] M. G. Andreassi and A. Della Corte , “Genetics of Bicuspid Aortic Valve Aortopathy,” Current Opinion in Cardiology 31, no. 6 (2016): 585–592. 27583373 10.1097/HCO.0000000000000328

[77] L. J. Martin , V. Pilipenko , K. M. Kaufman , et al., “Whole Exome Sequencing for Familial Bicuspid Aortic Valve Identifies Putative Variants,” Circulation: Cardiovascular Genetics 7, no. 5 (2014): 677–683. 25085919 10.1161/CIRCGENETICS.114.000526

[78] W. Sheng , D. Zhou , H. Dai , R. Zheng , A. Aihemaiti , and X. Liu , “Transcatheter Aortic Valve Replacement in Patients With Quadricuspid Aortic Valve: A Case Series and Systematic Review,” Cardiology Research and Practice 2025 (2025): 7815279. 39949952 10.1155/crp/7815279 PMC11824809

[79] P. Kantaputra , K. Buaban , N. Thongsee , et al., “Broad Spectrum of Anomalies Including Quadricuspid Aortic Valve Associated With a Novel Frameshift SALL4 Variant,” Clinical Genetics 104, no. 1 (2023): 133–135. 36756699 10.1111/cge.14307

