# Supplementary material for: First LDLRAP1 and Recurrent LDLR Mutations in Tunisian Families With Familial Hypercholesterolemia
Source: J Cell Mol Med. 2026 Jan 5;30(1):e70997. doi: 10.1111/jcmm.70997 (PMC12771596; doi:10.1111/jcmm.70997)
Supplement: Supplementary file 1 — Table S1: Primer sequences and corresponding annealing temperature. [file JCMM-30-e70997-s001.docx]

**Suppl. Table 1:** Primer sequences and corresponding annealing temperature

| **Gene** | **Forward Primer** | **Reverse Primer** | **Anneal Temperature** |
| --- | --- | --- | --- |
| ***LDLR*** | TCACTCCATCTCAAGCATCG  (exon 12) | CAACCAGTTTTCTGCGTTCA  (intron 12) | 55°C |
| ***LDLRAP1*** | GGATGCTGTTCAGCCTCAAG  (exon 2) | GCTGTAGCCACGATCCTCTT  (exon 2) | 58°C |
